# Analysis of Susceptibility to the Antimicrobial and Anti-Biofilm Activity of Human Milk Lactoferrin in Clinical Strains of *Streptococcus agalactiae* With Diverse Capsular and Sequence Types

**DOI:** 10.3389/fcimb.2021.740872

**Published:** 2021-09-20

**Authors:** Jacky Lu, Miriam A. Guevara, Jamisha D. Francis, Sabrina K. Spicer, Rebecca E. Moore, Schuyler A. Chambers, Kelly M. Craft, Shannon D. Manning, Steven D. Townsend, Jennifer A. Gaddy

**Affiliations:** ^1^Department of Pathology, Microbiology, and Immunology, Vanderbilt University School of Medicine, Nashville, TN, United States; ^2^Department of Chemistry, Vanderbilt University, Nashville, TN, United States; ^3^Department of Chemistry, Harvard University, Cambridge, MA, United States; ^4^Department of Microbiology and Molecular Genetics, Michigan State University, East Lansing, MI, United States; ^5^Division of Infectious Diseases, Department of Medicine, Vanderbilt University Medical Center, Nashville, TN, United States; ^6^Tennessee Valley Healthcare Systems, Department of Veterans Affairs, Nashville, TN, United States

**Keywords:** biofilm, lactoferrin, *Streptococcus agalactiae*, nutritional immunity, antimicrobial

## Abstract

Group B *Streptococcus* (GBS) is one of the leading infection-related causes of adverse maternal and neonatal outcomes. This includes chorioamnionitis, which leads to preterm ruptures of membranes and can ultimately result in preterm or stillbirth. Infection can also lead to maternal and neonatal sepsis that may contribute to mortality. Currently, treatment for GBS infection include a bolus of intrapartum antibiotic prophylaxis to mothers testing positive for GBS colonization during late pregnancy. Lactoferrin is an antimicrobial peptide expressed in human breast milk, mucosal epithelia, and secondary granules of neutrophils. We previously demonstrated that lactoferrin possesses antimicrobial and antibiofilm properties against several strains of GBS. This is largely due to the ability of lactoferrin to bind and sequester iron. We expanded upon that study by assessing the effects of purified human breast milk lactoferrin against a panel of phenotypically and genetically diverse isolates of GBS. Of the 25 GBS isolates screened, lactoferrin reduced bacterial growth in 14 and biofilm formation in 21 strains. Stratifying the data, we observed that colonizing strains were more susceptible to the growth inhibition activity of lactoferrin than invasive isolates at lactoferrin concentrations between 250-750 µg/mL. Treatment with 750 µg/mL of lactoferrin resulted in differences in bacterial growth and biofilm formation between discrete sequence types. Differences in bacterial growth were also observed between capsular serotypes 1a and III. Maternally isolated strains were more susceptible to lactoferrin with respect to bacterial growth, but not biofilm formation, compared to neonatal sepsis isolates. Finally, high biofilm forming GBS strains were more impacted by lactoferrin across all isolates tested. Taken together, this study demonstrates that lactoferrin possesses antimicrobial and antibiofilm properties against a wide range of GBS isolates, with maternally isolated colonizing strains being the most susceptible.

## Introduction

*Streptococcus agalactiae*, more commonly known as Group B *Streptococcus* (GBS), is amongst the leading infection-related causes of adverse pregnancy and neonatal outcomes (Shabayek and Spellerberg, 2018). Adverse maternal complications include chorioamnionitis, preterm premature rupture of membranes (PPROM), preterm birth, stillbirth, and maternal sepsis (Goldenberg et al., 2008; Koumans et al., 2012). For the newborn, GBS infections can result in early- and late- onset neonatal sepsis, meningitis, and endocarditis. Early onset disease (EOD) occurs in neonates up to a week after birth (Verani et al., 2010). Neonates with EOD usually present with pneumonia and sepsis. In contrast, late onset disease (LOD) defines infection between 1-week and 3 months after birth and most commonly manifests as sepsis and meningitis. Newborns who survive LOD frequently suffer from neurodevelopmental impairments (Russell et al., 2017).

GBS is a gram-positive encapsulated bacterium, and a commensal member of the human microflora in the gastrointestinal tract. While GBS asymptomatically colonizes 20-30% of adults, the bacterium may traverse from the lower gastrointestinal tract to the vagina and infect the neonate through ascending infection or ingestion/inhalation of infectious fluids during childbirth (Verani et al., 2010). Indeed, the primary risk factor for EOD is rectovaginal colonization of pregnant women with GBS during delivery (Seale et al., 2017b). The ability of GBS to colonize and persist in the maternal urogenital tract to cause disease is related to its ability to form biofilms (Rosini and Margarit, 2015). Colonization rates differ worldwide, spanning between 6.5-36% (Shabayek and Spellerberg, 2018). Some recent reports include colonization rates of 13.2% in a cohort in Ethiopia (Ali et al., 2020), 16.6% in the Western Cape region of South Africa (Africa and Kaambo, 2018), and 21.6% over a twelve-year span in North Carolina, USA (Edwards et al., 2019).

GBS strains can be divided into 10 distinct serotypes (Ia, Ib, and II to IX) based on a serological reaction directed against the polysaccharide capsule (Teatero et al., 2017). The streptococcal polysaccharide capsule facilitates evasion of the innate immune response by protecting the bacterial cell from deposition of complement, opsonization, and phagocytosis (Winkelstein et al., 1980; Brown et al., 1983; Abeyta et al., 2003). A recent study from our laboratory revealed that the GBS capsule aids in biofilm formation and ascending infection of the reproductive tract during pregnancy (Noble et al., 2021). Moreover, the capsule across all serotypes shares terminal sialic acid (Sia) residues that allow molecular mimicry of human cell surface sialic acids. This allows interaction with Sia-receptors, Siglecs, on innate immune cells that serve to dampen inflammatory responses (Carlin et al., 2007). Different capsular serotypes result in different range and severity in human disease. For instance, capsular serotype III strains are associated with higher rates of invasive neonatal disease (Alhhazmi et al., 2017) and account for the majority of late-onset meningitis cases in neonates (Bellais et al., 2012). In contrast, serotype Ia and V predominate among invasive isolates in non-pregnant cases (Phares et al., 2008). However, dominate serotypes fluctuate between regions and across time (Shabayek and Spellerberg, 2018).

Another method by which GBS strains are grouped and characterized is multi-locus sequence typing (MLST), which classifies strains into sequence types (STs) based on allelic variation within seven conserved housekeeping genes (Jones et al., 2003). Based on phylogenetic analysis, GBS STs can be grouped into multiple clonal complexes (CCs) with most human isolates belonging to CC1, CC10, CC17, CC19, CC23, and CC26 (Sørensen et al., 2010). Similar to capsular type, ST diversity also manifests in different disease outcomes and severities. For instance, a study with GBS from multiple continents revealed that STs 1 and 19 have been linked to asymptomatic colonization, while ST-17 predominately related to invasive neonatal disease. ST-23 was associated with both carriage and invasive GBS disease (Jones et al., 2003). All four STs, however, were found to colonize pregnant women at higher rates in different patient populations (Manning et al., 2009). ST-17 serotype III, alongside ST-291, belong to CC17, a group of GBS noted for its hypervirulence (Bellais et al., 2012). ST-17 is strongly linked to both EOD and LOD, as well as meningitis (Musser et al., 1989; Lin et al., 2006; Manning et al., 2008). Meanwhile, ST-291 belonging to serotype IV is strongly associated with EOD and septicemia (Tien et al., 2011; Wang et al., 2015).

In order to combat GBS, the immune system deploys a repertoire of antimicrobial peptides. These peptides aid in combatting infection through the process of nutritional immunity, or the sequestration of essential metals to starve bacteria (Hood and Skaar, 2012). Bacteria require these trace elements as cofactors for essential biological processes. One important example of a protein expressed in defense against GBS is lactoferrin (Kothary et al., 2017; Lu et al., 2020). Lactoferrin is a glycoprotein that contains two iron binding sites (Weinberg, 1975) and was shown to have antimicrobial activity against a wide range of bacterial, viral, and fungal pathogens (Lu et al., 2020). Indeed, our previous study demonstrated that human breast milk lactoferrin has antimicrobial and anti-biofilm activity against GBS and inhibits some GBS strains from adhering to human gestational membranes (Lu et al., 2021). In this study, we advanced our findings by analyzing the antimicrobial and anti-biofilm effects of lactoferrin against a larger panel of clinical GBS strains that vary by capsular serotype, ST, isolation source, and clinical presentation. We observed broad antimicrobial and antibiofilm action by lactoferrin against most GBS strains, though the maternal colonizing strains were more susceptible to inhibitory effects than the neonatal invasive strains.

## Materials and Methods

### Bacterial Strains and Culture Conditions

This study utilized a diverse set of 25 previously characterized *S. agalactiae* strains recovered from neonates with invasive disease (Manning et al., 2009) and colonized mothers sampled before and after childbirth (Manning et al., 2008); all strains were originally isolated by Dr. H. Dele Davies (Davies et al., 2001; Spaetgens et al., 2002). GBS strains were cultured on tryptic soy agar plates supplemented with 5% sheep blood (blood agar plates) at 37°C in ambient air overnight. Bacteria were sub-cultured from blood agar plates into liquid medium (Todd Hewitt Broth; THB) and incubated in aerobic conditions (ambient air, shaking at 200 rpm) at 37°C overnight. The following day, bacterial density was measured spectrophotometrically to determine the optical density at 600 nm (OD_600_). These bacterial cultures were used for growth, viability, biofilm, and co-culture assays.

### Purification of Lactoferrin From Human Breast Milk

Human lactoferrin was isolated from breast milk as previously described (Lu et al., 2021). Briefly, expressed human breast milk was gathered from 17 healthy donors between 3 days and 3 months post-partum and stored between -80 and -20˚C. De-identified human milk samples were provided by Dr. J. Hendrik Weitkamp from the Vanderbilt Department of Pediatrics, under a collection protocol approved by the Vanderbilt University Institutional Review Board (IRB #100897). Milk samples were thawed and centrifuged at 8000 g for 45 minutes to separate milk fats from the soluble fraction. Following centrifugation, the resultant top lipid layer was removed. Subsequently, proteins were precipitated from the soluble fraction by the addition of ammonium sulfate to the soluble fraction and incubation at 4˚C overnight. Precipitated proteins were fractionated by ion-exchange chromatography. Cation exchange (CM Sephadex C-50, GE Healthcare) resin suspension was packed in a column (300 x 18 mm). After sample loading, the column was washed with equilibration buffer until the absorbance at 280nm was less than 0.05. The bound protein was then displaced from the resin by a stepwise elution protocol. For elution, 10mM sodium phosphate buffer containing 0.4 M NaCl, 0.6 M NaCl and 0.8 M NaCl were used as elution buffer A, B, and C, respectively. First, elution buffer A was passed through the column. 5 mL fractions were collected and the OD_280_ value of each fraction was measured by a UV-vis spectrophotometer. The elution was continued until the fractions showed a minimum OD of 0.03. Further elution of the bound protein was carried out with elution buffer B and C. The Identity of the fractions were determined by high resolution mass spectrometry analysis. Fractions containing greater than 99% lactoferrin were combined and used in the assays. All lactoferrin used in this study was in the apo-form.

### Evaluation of Bacterial Growth

Bacterial growth was determined by a spectrophometric reading as previously described (Lu et al., 2021). Briefly, optical density measurements at 600 nm (OD_600_) were recorded to determine bacterial growth. GBS cultures were grown to stationary phase (OD_600_ between 0.2-0.3) and diluted at 1:10 in metal-limited THB medium (50% THB with 50% calprotectin buffer [100 mM NaCl, 3 mM CaCl_2_, 20 mM Tris pH 7.5 (Senkovich et al., 2010; Haley et al., 2015)]. 100 µL of 1:10 diluted cultures were added to each well in a 96-well plate. The appropriate concentration of purified lactoferrin (0, 250, 500, 750, or 1000 µg/mL, concentrations which are physiologically relevant to the host-pathogen *in vivo* environment) was added into each corresponding well. The plates were incubated at 37°C overnight. The following day, bacterial density was determined by measuring OD_600_.

### Quantification of Bacterial Biofilms

A crystal violet assay was utilized to evaluate bacterial biofilms as previously described (Gaddy et al., 2009; Lu et al., 2021). Briefly, overnight GBS cultures were diluted 1:10 in THB-CP medium in 96-well plates. To analyze the effect of lactoferrin on biofilm inhibition, lactoferrin was applied in increasing concentrations (0, 250, 500, 750, or 1000 µg/mL) at the time of inoculation. Biofilms were allowed to form at 37°C in ambient air overnight. OD_600_ was determined using a spectrophotometer and supernatant was removed and replaced with 0.1% crystal violet stain for thirty minutes. Wells were washed with deionized water three times and dried. The retained crystal violet was resolubilized with a solution of 80% ethanol and 20% acetone. Plates were incubated for at least 30 minutes and optical density was determined at 560 nm (OD_560_). Quantification was determined by using a ratio of OD_560_/OD_600_.

### Statistical Analyses

Statistical analyses of biofilm formation and bacterial growth were performed using Student’s t-test or a one-way ANOVA with either Tukey’s or Dunnett’s *post hoc* correction for multiple comparisons. All reported *P* values are adjusted to account for multiple comparisons. *P* values of ≤0.05 were considered significant. All data analyzed in this work were derived from at least three biological replicates, data points reflect mean of technical replicates (1-3 technical replicates per biological replicate). Statistical analyses were performed using GraphPad Prism software (Versions 6 and 9, GraphPad Prism Software Inc., La Jolla, California).

## Results

### Human Breast Milk Lactoferrin Suppresses Bacterial Growth in Many Clinical GBS Isolates

We previously reported that human breast milk lactoferrin possesses antimicrobial activity against three clinical isolates of GBS (Lu et al., 2021). To enhance the generalizability of these findings, we increased the number of GBS strains, thereby capturing more isolates across diverse capsular serotypes and STs. We investigated the effects of lactoferrin treatment across increasing concentrations against this panel of clinical isolates. Out of the 25 GBS strains screened, four strains exhibited inhibition of bacterial growth when treated with 250 µg/mL of human lactoferrin (**Table 1**; P < 0.05, Student’s t-test). Growth of 9 total strains was inhibited when treated with a concentration of lactoferrin of 500 µg/mL (P < 0.05, Student’s *t*-test). At 750 µg/mL of lactoferrin, 14 strains exhibited a decrease in bacterial growth as compared to media only (P < 0.05, Student’s *t*-test). Finally, the growth of 14 strains was inhibited when treated with 1,000 µg/mL of lactoferrin (P < 0.05, Student’s *t*-test). While there was a trending decrease in bacterial growth for 10 strains relative to the media control, the differences were not statistically significant. No significant differences in growth in medium alone were observed across diverse strains of GBS.

**Table 1:** Isolation source, capsular type, and sequence type (ST) of clinical strains of *Streptococcus agalactiae* used in this study and the minimum inhibitory concentration (MIC) of lactoferrin required to suppress growth (as determined by OD_600_) and biofilm (as determined by OD_560_/OD_600_).

Strain Number	Strain Type	Sequence Type	Capsular Serotype	Isolation Source	Growth MIC	Biofilm MIC
**GB0002**	Colonizing	ST-23	cpsIa	Vaginal/rectal colonization	250 µg/mL	250 µg/mL
**GB0012**	Colonizing	ST-1	cpsV	Vaginal/rectal colonization	750 µg/mL	250 µg/mL
**GB0037**	Invasive	ST-1	cpsV	EOD/sepsis	>1000 µg/mL	250 µg/mL
**GB0064**	Invasive	ST-17	cpsIII	EOD/sepsis	>1000 µg/mL	500 µg/mL
**GB0066**	Invasive	ST-19	cpsIII	EOD/sepsis	500 µg/mL	>1000 µg/mL
**GB0069**	Invasive	ST-17	cpsIII	EOD/sepsis	500 µg/mL	>1000 µg/mL
**GB0079**	Invasive	ST-19	cpsIII	EOD/sepsis	>1000 µg/mL	250 µg/mL
**GB0083**	Colonizing	ST-1	cpsVI	Vaginal/rectal colonization	>1000 µg/mL	250 µg/mL
**GB0112**	Colonizing	ST-12	cpsIII	Vaginal/rectal colonization	500 µg/mL	250 µg/mL
**GB0115**	Colonizing	ST-17	cpsIII	Vaginal/rectal colonization	250 µg/mL	<1000 µg/mL
**GB0241**	Colonizing	ST-23	cpsV	Vaginal/rectal colonization	>1000 µg/mL	250 µg/mL
**GB0285**	Colonizing	ST-12	cpsII	Vaginal/rectal colonization	750 µg/mL	250 µg/mL
**GB0291**	Colonizing	ST-12	cpsII	Vaginal/rectal colonization	500 µg/mL	250 µg/mL
**GB0374**	Invasive	ST-12	cpsIb	EOD/sepsis	>1000 µg/mL	250 µg/mL
**GB0377**	Invasive	ST-19	cpsIII	EOD/sepsis	>1000 µg/mL	250 µg/mL
**GB0390**	Invasive	ST-23	cpsIa	EOD/sepsis	>1000 µg/mL	250 µg/mL
**GB0397**	Invasive	ST-23	cpsIII	EOD/sepsis	750 µg/mL	250 µg/mL
**GB0411**	Invasive	ST-17	cpsIII	EOD/sepsis	750 µg/mL	<1000 µg/mL
**GB0418**	Invasive	ST-17	cpsIII	EOD/sepsis	750 µg/mL	250 µg/mL
**GB0438**	Invasive	ST-12	cpsIb	LOD/sepsis	>1000 µg/mL	250 µg/mL
**GB0571**	Colonizing	ST-19	cpsIII	Vaginal/rectal colonization	250 µg/mL	250 µg/mL
**GB0590**	Colonizing	ST-19	cpsIII	Vaginal/rectal colonization	>1000 µg/mL	250 µg/mL
**GB0653**	Colonizing	ST-12	cpsII	Vaginal/rectal colonization	500 µg/mL	250 µg/mL
**GB0654**	Colonizing	ST-17	cpsIII	Vaginal/rectal colonization	250 µg/mL	250 µg/mL
**GB0663**	Colonizing	ST-19	cpsIII	Vaginal/rectal colonization	>1000 µg/mL	250 µg/mL

### Human Breast Milk Lactoferrin Exhibits Anti-Biofilm Activity Against Numerous Clinical GBS Strains

Because we previously described the iron-dependent anti-biofilm properties of lactoferrin against a limited number of GBS strains (Lu et al., 2021), we expanded the number of GBS strains to determine if lactoferrin can suppress other strains with diverse genetic backgrounds. Among the 25 strains assayed, 20 strains exhibited a significant decrease in biofilm formation when treated with 250 µg/mL (**Table 1**; P < 0.05, Student’s t-test). When the concentration of lactoferrin was increased to 500 µg/mL, one additional strain showed susceptibility and a decrease in biofilm production. Of all the strains screened, only four had no reduction in biofilm formation across increasing concentrations of human lactoferrin.

### Colonizing GBS Strains Are More Susceptible to Lactoferrin Than Invasive Isolates

**Figure 1:**
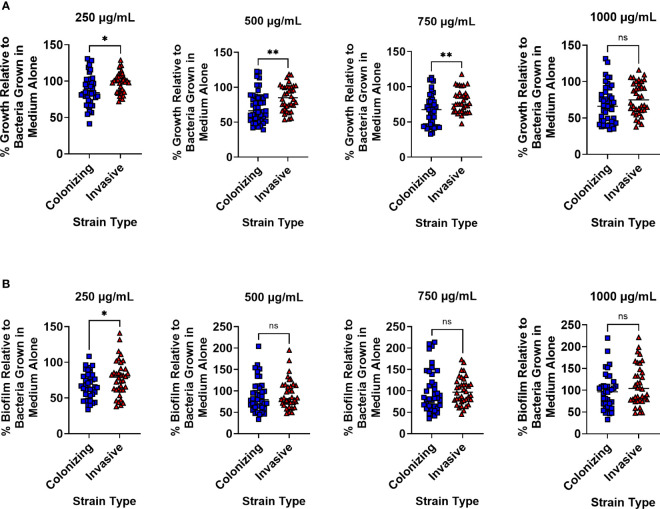
Analysis of susceptibility to lactoferrin-associated growth or biofilm inhibition in invasive *vs*. clinical isolates of Group B *Streptococcus* (GBS). GBS strains isolated from colonized patients, or patients experiencing invasive disease were grown in medium alone or increasing concentrations of lactoferrin. **(A)** Bacterial growth was measured at 24 hr post-inoculation and percent growth was calculated with reference to growth observed in the medium alone negative control. At 250, 500, and 750 µg/mL, colonizing strains of GBS (blue squares) exhibited greater growth inhibition than invasive strains (red triangles) as determined by Student’s t-test with Welch’s correction (*P < 0.05, and **P < 0.01). **(B)** Bacterial biofilm was measured at 24 hr post-inoculation and percent growth was calculated with reference to growth observed in the medium alone negative control. At 250 µg/mL, colonizing strains of GBS (blue squares) exhibited greater biofilm inhibition than invasive strains (red triangles) as determined by Student’s t-test with Welch’s correction (*P < 0.05). ns, not statistically significant.

GBS strains were grouped into two groups - colonizing and invasive – and susceptibility to lactoferrin was compared to GBS grown in medium alone without lactoferrin supplementation. At 250 µg/mL, the 13 colonizing strains were more susceptible to lactoferrin compared to the 12 invasive strains in respect to bacterial growth with a 12.21% *vs* 1.76% mean reduction, respectively (**Figure 1A**; P < 0.05, Student’s t-test). Increasing the concentration of lactoferrin to 500 µg/mL resulted in enhanced suppression of bacterial growth in the colonizing *versus* invasive strains (28.14% *vs* 17.48% mean reduction, respectively) (P < 0.01, Student’s t-test). Although the addition of 750 µg/mL of lactoferrin decreased bacterial growth for both types of strains, the invasive strains were more resistant to inhibition than the colonizing strains (20.71% *vs* 33.17% mean reduction, respectively) (P < 0.01, Student’s t-test). Lactoferrin at 1,000 µg/mL asserted antimicrobial activity against both types of strains but no differences were observed between the two strain types. With respect to biofilm formation, only the addition of lactoferrin at 250 µg/mL inhibited biofilm formation in the colonizing strains more than invasive ones (33.25% *vs* 22.01% mean reduction, respectively) (**Figure 1B**; P < 0.05, Student’s t-test).

### Treatment With Human Lactoferrin at 750 µg/mL Reveals Differences in Susceptibility Between GBS Sequence Types (STs)

**Figure 2:**
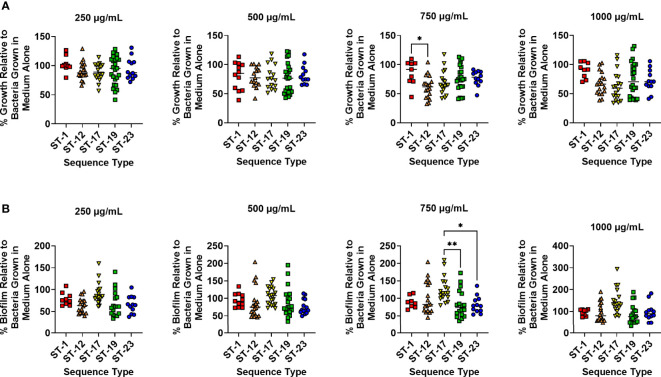
Analysis of susceptibility to lactoferrin-associated growth inhibition in diverse sequence types (STs) of Group B *Streptococcus* (GBS). GBS strains isolated with a variety of sequence type (ST-1, red; ST-12, orange; ST-17, yellow; ST-19, green; ST-23, blue) were grown in medium alone or increasing concentrations of lactoferrin. **(A)** Bacterial growth was measured at 24 hr post-inoculation and percent growth was calculated with reference to growth observed in the medium alone negative control. At 750 µg/mL, ST-12 strains of GBS exhibited greater growth inhibition than ST-1 strains as determined by one-way ANOVA with *post hoc* Tukey’s test (*P < 0.05). **(B)** Bacterial biofilm was measured at 24 hr post-inoculation and percent biofilm was calculated with reference to biofilm observed in the medium alone negative control. At 750 µg/mL, ST-19 and ST-23 strains of GBS exhibited greater biofilm inhibition than ST-17 strains as determined by one-way ANOVA with *post hoc* Tukey’s test (*P < 0.05, and **P < 0.01).

Because different GBS STs are associated with maternal colonization and neonatal disease, it is possible that different strains have variable mechanisms to cope with iron starvation. To investigate this possibility, GBS strains were binned by ST and susceptibility to lactoferrin was analyzed between STs. No significant differences in bacterial growth were detected with treatment at 250, 500, and 1,000 µg/mL of lactoferrin between the different STs; however, differences were observed with 750 µg/mL of lactoferrin. Specifically, the ST-1 strains were more resistant to bacterial growth suppression compared to ST-12 strains (**Figure 2A**; P < 0.05, One-way ANOVA; *post hoc* Tukey’s test). Similar differences in biofilm suppression were observed between STs while treating with 750 µg/mL of lactoferrin. At this concentration, the ST-17 strains were more resistant to the antibiofilm activity of lactoferrin compared to both ST-19 and ST-23 strains (**Figure 2B**; P < 0.01 and P < 0.05, respectively, One-way ANOVA; *post hoc* Tukey’s test).

### Treatment With Human Lactoferrin at 250 µg/mL Reveals Differences in Susceptibility Across Capsular Types

**Figure 3:**
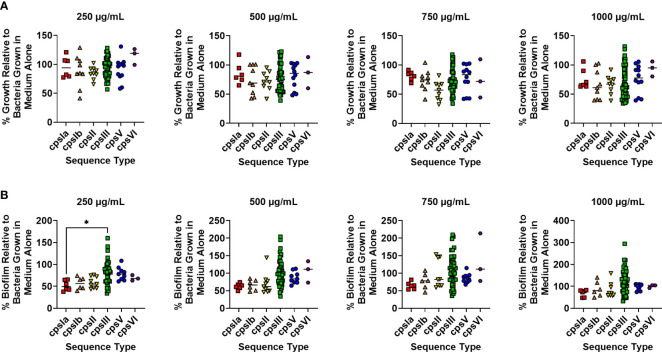
Analysis of susceptibility to lactoferrin-associated growth or biofilm inhibition in diverse capsular serotypes of Group B *Streptococcus* (GBS). GBS strains isolated with a span of capsular serotypes (cpsIa, red; cpsIb, orange; cpsII, yellow; cpsIII, green; cpsV, blue; cpsVI, purple) were grown in medium alone or increasing concentrations of lactoferrin. **(A)** Bacterial growth was measured at 24 hr post-inoculation and percent growth was calculated with reference to growth observed in the medium alone negative controls. No differences in growth were detected across molecular serotype as determined by one-way ANOVA with *post hoc* Tukey’s test (*P < 0.05). **(B)** Bacterial biofilm formation was measured at 24 hr post-inoculation and percent growth was calculated with reference to growth observed in the medium alone negative controls. At 250 µg/mL, cps1a strains of GBS exhibited greater biofilm inhibition than cpsIII strains as determined by one-way ANOVA with *post hoc* Tukey’s test (*P < 0.05).

It is plausible that capsular type may also influence resistance to lactoferrin. To test this, strains were grouped into cohorts based on capsular serotype and susceptibility to lactoferrin across capsule types were analyzed. No significant differences in resistance against the antimicrobial activity of lactoferrin were observed between capsular types across increasing concentrations of lactoferrin (**Figure 3A**; P > 0.05, One-way ANOVA; *post hoc* Tukey’s test). However, treatment with lactoferrin at 250 µg/mL showed that capsular type III strains exhibited resistance to its antibiofilm activity compared to capsular type 1a isolates (**Figure 3B**; P < 0.05, One-way ANOVA; *post hoc* Tukey’s test). This phenotype was ablated with the additional stress imposed by increasing concentrations of lactoferrin.

### Lactoferrin Asserts Anti-Biofilm Effects Against Both High and Low Biofilm Formers but Enhances Biofilm Formation in Low Biofilm Formers at Higher Concentrations

**Figure 4:**
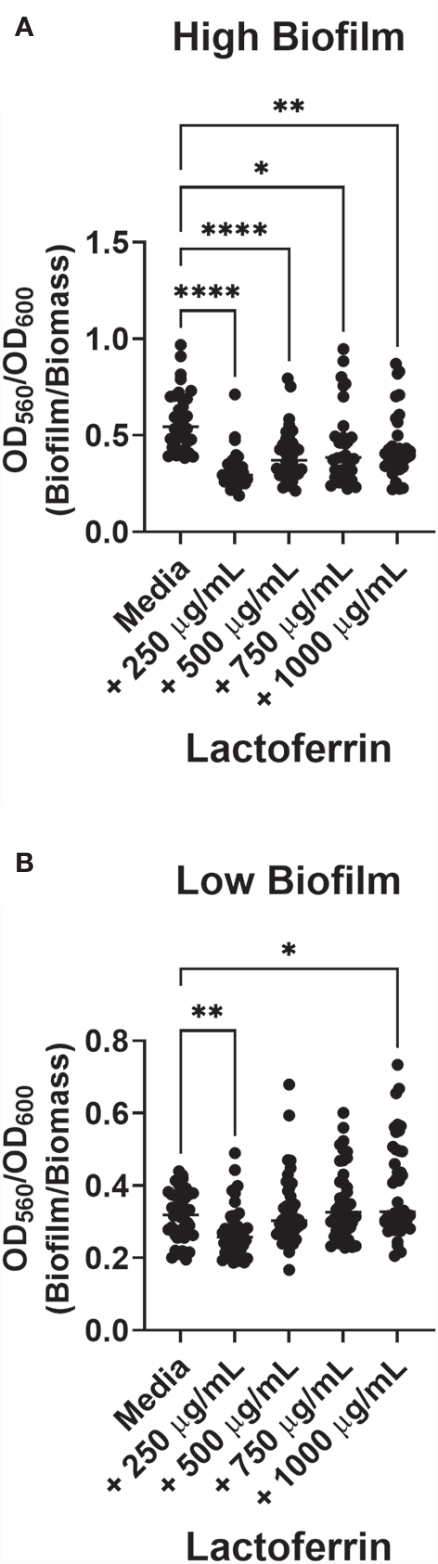
Lactoferrin-dependent inhibition of biofilm based on Group B *Streptococcus* (GBS) strain variation with respect to high or low biofilm production. GBS biofilm values (OD_560_) were pooled, and the geometric mean was determined. Strains were divided between high **(A)** and low **(B)** biofilm formers. High biofilm formers were more susceptible to biofilm inhibition by lactoferrin across all concentrations of lactoferrin. At 250 µg/mL, low biofilm forming strains of GBS exhibited inhibition of biofilm formation compared to media alone. However, treatment with 750 µg/mL and 1,000 µg/mL of lactoferrin resulted in an increase in biofilm formation, gas determined by Student’s t-test with Welch’s correction (*P < 0.05, **P < 0.01, ***P < 0.001, and ***P < 0.0001).

Because our group has previously observed a range of biofilm production across GBS strains (Parker et al., 2016), we sought to determine the geometric mean of biofilm produced by all isolates investigated in this study. GBS strains that form biofilms above the determined geometric mean (OD_560_/_600_ = 0.3965) were designated as “high” biofilm formers, while those below were named “low”. Treatment with 250 µg/mL of lactoferrin significantly inhibited biofilm formation in both high and low biofilm formers (**Figure 4**; P < 0.0001, Student’s t-test; P < 0.001, Student’s t-test, respectively), whereas 500 µg/mL of lactoferrin only inhibited the high biofilm formers (P < 0.0001, Student’s t-test). When treated with 750 µg/mL of lactoferrin, the high biofilm formers exhibited a decrease in biofilm formation (P < 0.05, Student’s t-test), however, the low biofilm formers (mean = 0.3183) showed increased biofilm production (mean = 0.3501) (P < 0.05, Student’s t-test). This discrepancy between high and low biofilm formers was further amplified at treatment with 1000 µg/mL of lactoferrin (mean = 0.3934) (P < 0.001, Student’s t-test).

## Discussion

In this present study, we expanded upon our previous work by increasing the panel of GBS strains to include phenotypically and genetically diverse clinical strains from diverse anatomical sites of isolation and assessing susceptibility to the antimicrobial and anti-biofilm activity of human milk lactoferrin. We discovered that lactoferrin possesses antimicrobial and antibiofilm properties against many diverse GBS strains. In particular, colonizing maternal strains were more susceptible to lactoferrin, compared to invasive neonatal strains.

Other studies have revealed that lactoferrin may contribute to improvement of reproductive tract infections and subsequent disease. For instance, vaginal lactoferrin supplementation in pregnant people with bacterial vaginosis reduced the rate of preterm birth (Miranda et al., 2019). Furthermore, other groups have identified lactoferrin as a critical component of cervicomucosal defense against a variety of lower genital tract infections caused by *Neisseria gonorrhoeae*, *Chlamydia trachomatis*, and *Trichomonas vaginalis* (Pino et al., 2017). One plausible explanation for this phenomenon may be that lactoferrin protects by repressing *Gardnerella*, *Prevotella*, and *Lachnospira* species within the host microbiome, thus decreasing competition for *Lactobacillus* species and allowing them to prevent dysbiosis by dominating the vaginal microbiota (Valenti et al., 2018). Adding credence to this possibility is that *Lactobacillus* species have been recognized as one of the few bacterial populations that lack a strict nutrient requirement for iron (Imbert and Blondeau, 1998). Thus, it is plausible that the antimicrobial activity of lactoferrin associated with its role in nutritional immunity would be largely ineffective against these important commensals. Indeed, our previous work has shown various Lactobacillus strains and their secreted products modulate GBS interactions with cells of the extraplacental membranes. Specifically, *Lactobacillus* supernatants inhibited GBS growth, biofilm formation and invasion of host cells, though strain-dependent effects were observed. Notably, supernatant from *L. reuteri* 6475 broadly inhibited growth in 36 distinct GBS strains and inhibited GBS growth to an average of 46.6% of each GBS strain alone (Shiroda et al., 2020). Thus, there is merit in studying the use of human lactoferrin alone or in combination with *Lactobacillus* spp. in the prevention of GBS-mediated preterm births and adverse pregnancy outcomes as maternal colonizing GBS strains can infect the fetus by ascending the gravid reproductive tract. Here, we have shown that maternal colonizing GBS strains are greatly susceptible to the antimicrobial and antibiofilm action of lactoferrin.

Our results indicate that certain STs are more susceptible than others at 750 µg/mL of lactoferrin treatment. The MLST scheme uses seven loci that encode enzymes involved in intermediary metabolism to distinguish GBS STs (Jones et al., 2003). Because iron is an important cofactor for many enzymes involved in bacterial metabolism and physiology (Andrews et al., 2003), lactoferrin can help defend against invading bacteria by starving the prokaryotic cells of nutritional iron needed for optimal enzyme activity. However, some enzymes are promiscuous with their utilization of transition metal co-factors. As a result, it is plausible that we witnessed variable antimicrobial effects of lactoferrin because some of these housekeeping enzymes, or another enzyme up- or downstream of its respective pathways, require iron for full function while others may use other transitional metals under conditions of iron starvation (Foster et al., 2014). An alternate explanation is that other enzymes in some pathways with similar functions may be able to compensate for the absence of or limited iron-cofactors. Perhaps the number of loci used in the MSTL were too limited. It is plausible that the effect of lactoferrin is indirect and does not involve the specific genes loci used in the MSTL. Further examination at the whole genome phylogeny may better differentiate lactoferrin effects across close or distantly related genomes regardless of cps type or isolation source.

The capsule of GBS plays an important role in pathogenesis in humans (Vornhagen et al., 2017). As different capsular types have been correlated to varying disease outcomes and susceptibility to antimicrobial agents (Teatero et al., 2017), we hypothesized that different capsular serotypes might have variable responses to the antimicrobial activity of lactoferrin. We observed differences in biofilm formation but not bacterial growth between different GBS capsular types under a lower treatment of lactoferrin. Given that the capsule is an important virulence factor for pathogenesis and evasion of immune assault (Gendrin et al., 2018), it was not surprising that no differences were observed in our controlled *in vitro* studies free of immune stressors. However, we did observe differences in GBS biofilm formation. Our group previously described the role of capsule in biofilm formation in GBS (Noble et al., 2021), further bolstering our findings. It is plausible that iron starvation alters capsule-mediated biofilm formation in certain capsular types of GBS. Further *in vitro* work with host cells or *in vivo* experiments are needed to bridge our gap in knowledge of these relationships between capsular serotype and lactoferrin.

In our study, we further analyzed differences in biofilm inhibition by lactoferrin between strains that formed robust biofilms compared to those that formed low or weak biofilms. Lactoferrin inhibited biofilm formation in high biofilm forming isolates, which is consistent to with our previous study in a smaller cohort of strains (Lu et al., 2021). The intersection between iron and biofilm formation has also been studied in other bacterial pathogens. One study of interest by Trappetti and colleagues described the role of the mononuclear iron protein S-ribosylhomocysteine lyase (LuxS) in quorum sensing and biofilm formation in *Streptococcus pneumoniae* (Trappetti et al., 2011). Consistent with their work, we also observed that iron starvation results in inhibition of biofilm formation. Thus, it is plausible that GBS possesses similar iron-sensing pathways that govern biofilm formation. More work, however, is needed to elucidate and confirm the function of these pathways.

Colonization of the maternal genitourinary tract is the most important risk factor for neonatal GBS disease (Benitz et al., 1999). In a longitudinal study performed by Kwatra and colleagues, up to 50% of the study cohort was transiently colonized by GBS at some point during pregnancy, highlighting the dynamic nature of GBS colonization (Kwatra et al., 2014). Currently in the United States, pregnant individuals are screened for the presence of GBS between 35 and 37 weeks of gestation (Carrillo-Ávila et al., 2018). If a patient tests positive for GBS, then intrapartum antibiotic prophylaxis (IAP) is administered during labor and delivery to prevent neonatal EOD (Seale et al., 2017a). Though antibiotic treatment is the only current preventative strategy available, the efficacy of IAP against EOD is around 80% (Fairlie et al., 2013). Despite the availability of IAP, however, the rates of LOD have remained unchanged (Nanduri et al., 2019). Additional drawbacks associated with the use of IAP (Rao and Khanna, 2020) include hypersensitivities to first-line antibiotics (Matteson et al., 2008), alteration of the neonatal microbiota (Nogacka et al., 2017), and emergence of antibiotic resistant strains (Hayes et al., 2020). Hence, the discovery of alternative therapies is critical and may help overcome these IAP drawbacks. Breastfeeding has been associated with protection against infection and could be exploited as a risk-mitigation strategy. However, cases of GBS transfer by breast milk have been recorded (Ager et al., 2020). The protective effects of breast milk are likely derived from the antimicrobial and immunomodulatory molecules that comprise milk, including lactoferrin and human milk oligosaccharides (Andreas et al., 2015). Thus, utilization of these specific molecules could be leveraged to mitigate the risk of GBS transmission. In the study herein, we found that maternal colonizing GBS strains are most susceptible to lactoferrin, suggesting that the antimicrobial peptide may be a viable candidate to aid in the prevention of GBS disease.

## Data Availability Statement

The original contributions presented in the study are included in the article/supplementary material. Further inquiries can be directed to the corresponding authors.

## Ethics Statement

Ethics approval to carry out this study was provided by the Vanderbilt University Institutional Review Board (IRB #100897 for human milk donation).

## Author Contributions

KC, JL, and ST purified lactoferrin for the studies. JL performed bacterial culture experiments. SDM curated and validated the clinical strains for use in this study. JL, JF, MG, SC, RM, SS, KC, ST, and JG conceptualized and analyzed results and interpreted data, and wrote and edited the manuscript for critical content. All authors contributed to the article and approved the submitted version.

## Funding

This work was funded by the National Institutes of Health grants HD090061 (to JG) and the Department of Veterans Affairs Office of Research BX005352 (to JG). which supported wet bench experiments, 2T32HL007411-39 (to JL), 2T32AI112541-06 (to JF), which supported their time and efforts, National Science Foundation 1847804 which supported reagents and wet bench work. Additional support was provided by the Vanderbilt Institute for Clinical and Translational Research program supported by the National Center for Research Resources, Grant UL1 RR024975-01, and the National Center for Advancing Translational Sciences, Grant 2 UL1 TR000445-06 which supports access to Core Facilities and Biostatistics support.

## Conflict of Interest

The authors declare that the research was conducted in the absence of any commercial or financial relationships that could be construed as a potential conflict of interest.

## Publisher’s Note

All claims expressed in this article are solely those of the authors and do not necessarily represent those of their affiliated organizations, or those of the publisher, the editors and the reviewers. Any product that may be evaluated in this article, or claim that may be made by its manufacturer, is not guaranteed or endorsed by the publisher.
